# Alcohol consumption patterns of the Hungarian general and Roma populations

**DOI:** 10.3389/fpubh.2022.1003129

**Published:** 2023-01-10

**Authors:** Ali Abbas Mohammad Kurshed, Ferenc Vincze, Péter Pikó, Zsigmond Kósa, János Sándor, Róza Ádány, Judit Diószegi

**Affiliations:** ^1^Department of Public Health and Epidemiology, Faculty of Medicine, University of Debrecen, Debrecen, Hungary; ^2^Doctoral School of Health Sciences, University of Debrecen, Debrecen, Hungary; ^3^Institute of Nutrition and Food Science, University of Dhaka, Dhaka, Bangladesh; ^4^ELKH-DE Public Health Research Group, Department of Public Health and Epidemiology, Faculty of Medicine University of Debrecen, Debrecen, Hungary; ^5^Department of Health Methodology and Public Health, Faculty of Health, University of Debrecen, Nyíregyháza, Hungary

**Keywords:** alcohol consumption, AUDIT, Roma population, Hungarian population, decomposition

## Abstract

**Introduction:**

Harmful alcohol use is a significant public health problem worldwide, though the alcohol-related burden affects disproportionately certain populations and ethnic minorities, with the WHO European Region being the most heavily affected and putting an increased risk on Roma populations. This ethnic minority group is the largest and most vulnerable ethnic minority in Europe and Hungary as well.

**Methods:**

The present study aims to describe and compare the alcohol consumption behaviors of the Hungarian general and Roma populations using the Alcohol Use Disorders Identification Test (AUDIT), which provides a comprehensive view of alcohol consumption behavior. In addition, a decomposition analysis was performed when the multivariate logistic or Poisson regression model showed significant differences between the two samples.

**Results:**

Our findings suggest that Roma people in our study sample experience more alcohol-related harm, even when considering past problems. The decomposition analysis revealed that gender and relationship status differences act more intensely among Roma than non-Roma when considering alcohol-related harm.

**Discussion:**

Equalizing these differences would be expected to reduce the Hungarian general and Roma populations' alcohol-related harm frequency gap. Investigating alcohol-attributed harms at the ethnicity level provides important information to identify high-risk groups and, thus, to design and implement more targeted and accessible interventions for alcohol problems.

## 1. Introduction

Globally, alcohol consumption has been considered one of the leading risk factors for illness and mortality (1), ultimately contributing to increased premature death and the loss of healthy life years (2). According to estimates, harmful alcohol consumption accounted for 1.78 million deaths worldwide in 2020 (3); moreover, research suggests that during the COVID-19 pandemic, alcohol consumption and related harm and deaths increased further (4, 5). Both the proportion of alcohol-attributable deaths and DALYs (10.1% of all deaths and 10.8% of all DALYs) and consumption levels (9.8 l per capita in 2016) were found to be the highest in the WHO European Region (6). Although pure alcohol consumption decreased in most EU countries and also in Hungary (from 12.1 l in 2010 to 11.4 l in 2016; 19.1 liters for men and 4.5 l for women) (6), heavy alcohol use and related problems remained an issue (7), with Hungary having a significantly higher prevalence of alcohol use disorders (21.2% in Hungary vs. 8.8% in Europe) and alcohol dependence (9.4% in Hungary vs. 3.7% in Europe) compared to the average of the WHO European Region (6). According to sales data in 2019, Hungary was still characterized by consumption levels above the average of OECD countries (8.7 liters) and being among those countries (Latvia, followed by the Czech Republic, Austria, France, Hungary, Lithuania, and Slovenia) that reported consumption over 11 l per person (8).

Health behavior, including alcohol consumption patterns, may vary not only across populations but also among ethnicities (9). Previous research on racial and ethnic disparities in health behaviors consistently indicated unfavorable differences for obesity and leisure-time physical inactivity when comparing black and Mexican-American women with white women, and for smoking and physical inactivity in the case of black men (10). Meanwhile, results of studies on alcohol consumption in the United States are contradictory in terms of ethnic and racial comparisons (11–18). In Europe, the alcohol-related burden affects certain groups unevenly. Inequities have been identified regarding gender, education level, socioeconomic status, place of residence, and ethnicity. The extent of the effect and the complex interplay of these factors may vary across countries, potentially leading to differences in risk factors and consequences (9). Numerous studies have been conducted in European countries to compare the patterns of alcohol consumption behavior of Roma, the largest ethnic minority population, to that of the majority populations. Due to the lack of official documentation, fear of stigmatization, and reluctance to self-identify, the actual number of the Roma population remained unspecified, but it has been estimated that around 10–12 million Roma individuals inhabit the European Region (19), and the majority of them live in Central and Eastern Europe, representing more than 5% of the total population (20). In Hungary, the Roma represent 8.9% of the total population, with an estimated 876.000 individuals—a number that is steadily increasing (21). All over Europe, this minority population has faced decades of discrimination, which has manifested as marginalization in the formal labor market, poor education, inadequate access to healthcare services, and a less favorable health status compared to majority populations (22–29). Despite linguistic assimilation, the cultural identity and traditions maintained by Roma populations may still have a significant impact on their health behaviors.

In Central and Eastern Europe (CEE), Roma can be considered “perennial” outsiders, as described by Powell and Lever (30), Toma and Fosztó (31), and van Baar et al. (32). Roma disidentification and stigmatization have persisted over time, facilitating their social and spatial marginalization and giving rise to segregation (31). Negative stereotypes have been attached to Roma people, which do not seem to change with time (31, 32). In Romania, the Roma can be characterized by a history of political, social, and economic marginalization (33) and were even afflicted by slavery during history (34). Not much improvement could be observed over time, with Roma still being at the bottom of Romanian society (35), and unfavorable perceptions of Roma by the public also persisting (34, 36). Even in regions without severe spatial segregation, Roma communities experienced anti-Roma prejudice (31, 34). Roma still have been negatively stereotyped, as demonstrated in a study conducted in 2010 on the stereotyped ideas of Czechs about the Roma culture and lifestyle (37). Long-term discrimination and stigmatization may affect emotions and manifest in feelings of inferiority, potentially further enforcing separation, according to the authors Cretan et al. (36).

The situation in Hungary is not different from other CEE countries. According to results obtained in a study, spatial segregation and stigmatization (mainly associated with physical appearance and illegal activities) of Roma still persist (38, 39). In certain regions of the country (i.e., northeastern and south-western Hungary), even entire villages are segregated (40). Research in two segregated, urban Roma communities in Szeged (the third largest city in Hungary by population) pointed out that segregated Roma communities suffer injustice in three essential areas as a result of a combination of environmental and social injustice: access to work and goods, access to decent-quality housing, and access to essential public services due to financial and infrastructural difficulties. The majority of Roma have no other choice but to accept irregular, seasonal, low-waged, and often semi-illegal labor. All these challenges have a complex impact on the situation of the Roma people in Hungary (33, 39).

One study on alcohol intake conducted in Slovakia did not find any differences in overall consumption among men of Roma and non-Roma populations but identified lower rates in Roma women compared to non-Roma (41). On the other hand, Roma mothers had a higher risk of drinking alcohol during pregnancy than non-Roma mothers in this country (42). A study in Moldova presented the finding that Roma families spent more (116% of the non-Roma) on alcohol and tobacco compared to non-Roma (43). Roma people in Turkey could be characterized by a higher alcohol intake, and the frequency of alcoholism was 3.2 times higher among them than in others (44). In comparison with non-Roma, a higher proportion of Roma children were found to be daily alcohol users in Lithuania and Latvia, though the differences were not statistically significant (45). Results of a study conducted by Roma social workers in the Czech Republic showed that substance use, including regular excessive alcohol intake, was 2–6 times higher among Roma than the general population (46). Similarly, findings obtained from a study in Spain demonstrated that Roma women had significantly higher consumption of alcohol compared to non-Roma women (47). However, gender-specific results were obtained from a preceding study, where young Spanish Roma men were more likely to drink alcohol compared to other young men, whereas among women, alcohol consumption was less frequent among Roma than in the general population (48). Comparing the alcohol consumption habits of the Roma and non-Roma populations in Slovenia using the Alcohol Use Disorders Identification Test (AUDIT) revealed that although the Roma scored lower overall on the AUDIT and were characterized by a higher proportion of teetotalers, they also had a lower proportion of non-hazardous drinkers (49).

Although one study in Hungary found the prevalence of abstainers among Roma to be higher compared to the general adult population (29), alcohol consumption patterns among children and adolescents were less favorable compared to non-Roma (prevalence of daily alcohol consumption and drunkenness, lifetime prevalence of alcohol intoxication, earlier initiation of alcohol consumption) according to research results (50–52). Furthermore, the study on the decade of Roma inclusion identified negative changes among Hungarian Roma regarding heavy drinking, and the gap widened in comparison with the general population (53).

As demonstrated above, several studies have investigated the alcohol consumption behavior of Roma populations in Europe and Hungary from various aspects, and therefore using different alcohol consumption descriptions and assessment methods. Not only quantity but drinking frequency and intensity should also be considered when measuring the extent of harmful consumption (7). The AUDIT (Alcohol Use Disorder Identification Test) tool allows for the collection of information on various aspects of alcohol consumption and thus provides a comprehensive view of different dimensions of alcohol drinking behaviors, including alcohol-related harm, even when past problems are taken into account, which is also important when considering alcohol-related burden (54). Still, only one study in Slovenia (49) and none in Hungary collected information on alcohol consumption using the AUDIT, which provides a comprehensive view of different dimensions of alcohol consumption, drinking behaviors, and alcohol-related problems. By considering these, the present study aims to describe alcohol consumption behaviors of the Hungarian general (HG) population from an international point of view and also to compare them to those of the Hungarian Roma (HR) population using the AUDIT screening tool, which could be an important step when examining and addressing inequalities in alcohol-related harm from a national perspective (9). Data collection at the ethnicity level is an essential step in increasing knowledge about potential differences in alcohol consumption between Roma and non-Roma populations and also in understanding how ethnic inequities and inequalities act in addition to gender and other socioeconomic differences. Decomposing these differences may aid in the planning and implementation of targeted interventions aiming to reduce alcohol-related harm.

## 2. Materials and methods

### 2.1. Study design and sampling

Data used in this study were derived from a complex comparative health survey (55). This cross-sectional study was conducted in two counties (Borsod-Abaúj-Zemplén and Szabolcs-Szatmár-Bereg) of northeastern Hungary, where the majority of the Roma in the country live. The planned sample size of the study was 1,000 respondents, and probability sampling techniques were used to pick 500 subject representatives of HG and 500 subject representatives of HR living in segregated colonies. The assessment of the health behavior and health status of the study populations was based on three pillars: (i) physical examination (weight, height, waist circumference, blood pressure measurements, visual acuity, cardiovascular fitness tests, and measurements of the lateral spinal flexion and extensibility of the ischiocrural muscles) carried out in general practitioners' (GPs') offices; (ii) blood sample collection (for genetic analysis, routine laboratory investigations, and a lipid hormone profile); (iii) questionnaire surveys. Questionnaires were administered by trained practice nurses and Roma university students under the supervision of public health coordinators in the HG and HR populations, respectively. Data collection was carried out between 17 May and 29 August 2018. The study was approved by the Ethical Committee of the Hungarian Scientific Council on Health (61327-2017/EKU). Written informed consent was obtained from all participants in each study population in accordance with the Declaration of Helsinki.

#### 2.1.1. A sample representative of the Hungarian general population living in northeast Hungary

Study subjects from the Hungarian general population were recruited through a population-based disease registry called the General Practitioners' Morbidity Sentinel Stations Programme (GPMSSP), which program was founded in 1998 to monitor the incidence and prevalence of chronic non-communicable diseases of great public health importance. The source population of the GPMSSP includes all Hungarian citizens registered by the 59 participating general practitioners (56, 57). Our study population was randomly selected from the GPMSSP registry. Individuals 20–64 years of age registered by the participating general practitioners (GPs) of the two counties of northeast Hungary and living in private households were randomly enrolled. The desired sample size was 25 subjects from each of the 20 randomly selected GP practices in these two counties. As two GPs declined to participate, the final sample consisted of 450 participants from the practices of eighteen GPs. Data collection on health behavior was carried out in GP's practices during a health visit when questionnaires were delivered in a face-to-face manner by practice nurses.

#### 2.1.2. A sample representative of the Hungarian Roma population living in segregated colonies in northeast Hungary

A stratified multistep random sampling technique was applied to recruit Roma participants from the same counties in northeast Hungary (Hajdú-Bihar and Szabolcs-Szamár-Bereg counties). During a previous environmental survey, segregated colonies having more than 100 inhabitants were identified by Roma field workers whose ethnicity was confirmed by self-declaration (58). Following the necessary verification of this previously created database, 20 colonies were randomly selected, and 25 households were randomly chosen from each colony. From each household, one individual between the ages of 20 and 64 was enrolled by using a random table, which resulted in 500 sampled individuals. To overcome the potential difficulties and distrust of Roma individuals toward interviewers, questionnaires on health behavior were taken by trained Roma university students who were familiar with local and ethnic circumstances.

### 2.2. Studied variables

#### 2.2.1. Sociodemographic patterns

Questions about assessing sociodemographic characteristics were taken from the Hungarian version of the 2014 European Health Interview Survey (59). Respondents were classified according to covariate variables such as age, gender, marital status, highest level of education, economic activity, self-perceived financial status, and ethnicity. The following age groups were used: 20–34, 35–49, and 50+ years. The highest level of education was categorized as primary or less, secondary, high school, and tertiary school. We classified the respondents according to marital status as married, single, widowed, or divorced. Economic activity was described with the categories worker, unemployed, and pensioner/other allowance/student. Self-perceived financial status was measured by a standard question, with respondents assessing their prosperity on a five-point Likert scale from very bad to very good. Responses were categorized as good, satisfactory, and bad.

#### 2.2.2. Assessment of alcohol consumption patterns

Unhealthy alcohol consumption-related behaviors were assessed using the 10-item AUDIT (total score range of 0–40 points), which tool covers all the necessary domains of alcohol consumption to be examined (54) and has also been translated and validated in Hungarian (60). In line with the aims, (1) unhealthy alcohol behavior, (2) problematic drinking, (3) hazardous levels of alcohol use, (4) alcohol dependence, (5) alcohol-related harm, and (6) past alcohol problems were the primary outcome indicators. Based on the previously conducted international studies, problematic drinking was evaluated according to the 10-item AUDIT total score achievement according to the following thresholds: ≥8 points for men and ≥5 points for women (54, 61, 62). In our analyses, the criteria for possible hazardous alcohol consumption levels were met if a score of 1 or more on question 2 or question 3 was reached, while points scored above 0 on questions 4–6 were considered alcohol dependence symptoms. If at least one point was scored on questions 7–10, this indicated that alcohol-related harm was already being experienced by the respondents. The answers “Yes, but not in the last year” and “Yes, during the last year” to the final two AUDIT questions can also be examined individually from the overall score as evidence of past alcohol-related problems (54). Interviewers were trained prior to the study, and the questionnaire was delivered face-to-face.

### 2.3. Statistical analysis

First, the crude and age-adjusted frequencies of alcohol-related behaviors were calculated for the HR and HG samples. The association with the Roma ethnicity was evaluated by chi-square and Fisher's exact tests. Then, we used multiple Poisson and logistic regression models to investigate the influence of Roma ethnicity, independent of sociodemographic factors (age, gender, education, economic activity, marital status, and financial status), on the six studied primary outcome indicators. Associations were quantified by odds ratios (OR) and corresponding 95% confidence intervals (CIs).

We employed the Oaxaca–Blinder decomposition technique to explain the proportion of ethnic inequalities in alcohol consumption behaviors that could be explained by different socioeconomic variables. This regression-based counterfactual method was originally developed by Oaxaca (63) and Blinder (64) for linear models, but it is also possible to generalize the method for non-linear response models. This technique divides the gap between the mean values of an outcome into two components. The endowment component arises because of differences in the groups' characteristics; the coefficient component is attributed to different influences of these characteristics in each group. We performed the decomposition only when the multivariate logistic or Poisson regression model showed significant differences between the Hungarian general and Roma samples. Using the method described by Powers (65), the “mvdcmp” command was run in version 13 of the Stata software (Stata Corporation, College Station, Texas).

## 3. Results

### 3.1. Sociodemographic characteristics of the studied samples

The questionnaires were completed by 797 individuals (men: 35.26%, women: 64.74%). The proportion of people aged 50 and up was 35.13%. The majority of the respondents had primary education or less (51.82%) and lived with someone else (married: 63.86%). More than half of the population studied (66.62%) was an economically active worker, and the percentage of adults with satisfactory self-perceived economic status was 54.45%. The education attainment, economic activity, financial status, and gender variables indicated the less favorable status of the Roma respondents in comparison to the Hungarian general sample (Table 1).

**Table 1:** Characteristics of the Hungarian general and Roma populations.

**Characteristics**	**Variable**	**Total *n* (%)**	**HG *n* (%)**	**HR *n* (%)**	*P*-value^**^
Education^*^	Primary or less	413 (51.82%)	86 (20.98%)	327 (84.50%)	**< 0.001**
	Secondary	151 (18.95%)	109 (26.59%)	42 (10.85%)	
	High school	150 (18.82%)	138 (33.66%)	12 (3.10%)	
	Tertiary education	76 (9.54%)	75 (18.29%)	1 (0.26%)	
Economic activity^*^	Worker	531 (66.62%)	303 (73.90%)	228 (58.91%)	**< 0.001**
	Pensioner, other allowance, student	138 (17.31%)	69 (16.83%)	69 (17.83%)	
	Unemployed	115 (14.43%)	32 (7.80%)	83 (21.45%)	
Marital status^*^	Married	509 (63.86%)	253 (61.71%)	256 (66.15%)	0.240
	Single	166 (20.83%)	95 (23.17%)	71 (18.35%)	
	Widow, divorced	115 (14.43%)	60 (14.63%)	55 (14.21%)	
Financial status^*^	Good	185 (23.21%)	127 (30.98%)	58 (14.99%)	**< 0.001**
	Satisfactory	434 (54.45%)	227 (55.37%)	207 (53.49%)	
	Bad	165 (20.70%)	48 (11.71%)	117 (30.23%)	
Age category (years)	20–34	209 (26.22%)	99 (24.15%)	110 (28.42%)	0.348
	35–49	308 (38.64%)	160 (39.02%)	148 (38.24%)	
	50+	280 (35.13%)	151 (36.83%)	129 (33.33%)	
Gender	Men	281 (35.26%)	180 (43.90%)	101 (26.10%)	**< 0.001**
	Women	516 (64.74%)	230 (56.10%)	286 (73.90%)	
Total		797 (100%)	410 (100.00%)	387 (100.00%)	

HG, Hungarian general population; HR, Hungarian Roma population. ^*^Unequal to 100% due to missing cases (range of the missing answers proportion: 0.49–1.95%). ^**^Pearson's chi-squared and Fisher's exact test. Bold indicates statistical significance.

### 3.2. Crude and gender-adjusted alcohol consumption behaviors

Table 2 summarizes the crude gender and ethnic differences in alcohol consumption frequency according to AUDIT question 1. The crude frequency of 2–3 times a week or more was significantly lower among the HR sample (5.47%) than in the non-Roma sample (12.75%). After the gender stratification, the differences remained significant and also indicated a lower frequency of alcohol consumption among the HR sample. The differences in the frequency of consuming six or more drinks per occasion by gender between the HG and HR samples were insignificant (Table 3). The number of drinks consumed each week (based on the second question of AUDIT) differed significantly only between HG and HR women (*p* = 0.008). Drinking more than two drinks per week was found to be higher in HG men compared to women (28.5 and 6.99%, respectively, not presented in a table format).

**Table 2:** Drinking frequency and prevalence (AUDIT question 1) among the Hungarian general and Roma populations, by gender.

	**Never *n* (%)**	**Monthly or less *n* (%)**	**2–4 times a month *n* (%)**	**2–3 times a week or more *n* (%)**	*P*-value^*^
HG men	52 (29.05%)	55 (30.73%)	28 (15.64%)	44 (24.58%)	**0.007**
HR men	22 (21.78%)	52 (51.49%)	12 (11.88%)	15 (14.85%)	
HG women	139 (60.70%)	61 (26.64%)	21 (9.17%)	8 (3.49%)	**< 0.001**
HR women	180 (63.60%)	93 (32.86%)	4 (1.41%)	6 (2.12%)	
HG total	191 (46.81%)	116 (28.43%)	49 (12.01%)	52 (12.75%)	**< 0.001**
HR total	202 (52.60%)	145 (37.76%)	16 (4.17%)	21 (5.47%)	

HG, Hungarian general population; HR, Hungarian Roma population. ^*^Pearson's chi-squared and Fisher's exact test. Bold indicates statistical significance.

**Table 3:** The prevalence of consuming six or more drinks of alcohol per occasion (AUDIT question 3) among the Hungarian general and Roma populations, by gender.

	**Never *n* (%)**	**Less than monthly *n* (%)**	**Monthly or more often *n* (%)**	*p*-value^*^
HG men	118 (65.56%)	43 (23.89%)	19 (10.56%)	0.872
HR men	62 (62.63%)	25 (25.25%)	12 (12.12%)	
HG women	214 (93.04%)	14 (6.09%)	2 (0.87%)	0.342
HR women	259 (91.52%)	16 (5.65%)	8 (2.83%)	
HG total	332 (80.98%)	57 (13.90%)	21 (5.12%)	0.400
HR total	321 (84.03%)	41 (10.73%)	20 (5.24%)	

HG, Hungarian general population; HR, Hungarian Roma population. ^*^Pearson's chi-squared and Fisher's exact test.

In the Roma sample, the crude frequency of alcohol-related harm was significantly higher (13.33%) compared to the Hungarian general sample (6.20%) (Table 4). After the gender stratification, the alcohol-related harm frequency was three times higher among Roma men (HG: 12.92%, HR: 30.69%) and approximately seven times higher among Roma women (HG: 0.89, HR: 6.93%). Past alcohol problems were observed to be relatively low (1.71%) in Hungarian general respondents, while four times (4.95%) higher frequencies were assessed among the Roma population. 12.87% of the Roma men and 2.12% of the Roma women had past alcohol problems, according to the final two AUDIT questions; meanwhile, these frequencies were significantly lower among the Hungarian general respondents (men: 3.89%, women: 0.00%). The differences in problematic drinking, hazardous consumption, and alcohol dependence between the two studied samples were not significant.

**Table 4:** Alcohol consumption behaviors of the Hungarian general and Roma populations.

	**HG *n* (%)**	**HR *n* (%)**	*P*-value^*^
**Problematic drinking**			
Men	13 (7.39%)	14 (14.74%)	0.054
Women	4 (1.79%)	13 (4.78%)	0.084
Total	17 (4.25%)	27 (7.36%)	0.065
**Consumption at a hazardous level**			
Men	77 (43.02%)	46 (47.42%)	0.482
Women	25 (10.92%)	43 (15.30%)	0.147
Total	102 (25.00%)	89 (23.54%)	0.635
**Alcohol dependence**			
Men	14 (7.82%)	14 (14.14%)	0.094
Women	1 (0.44%)	6 (2.19%)	0.134
Total	15 (3.70%)	20 (5.36%)	0.265
**Alcohol-related harm**			
Men	23 (12.92%)	31 (30.69%)	**< 0.001**
Women	2 (0.89%)	19 (6.93%)	**0.001**
Total	25 (6.20%)	50 (13.33%)	**0.001**
**Past problems**			
Men	7 (3.89%)	13 (12.87%)	**0.007**
Women	0 (0.00%)	6 (2.12%)	**0.035**
Total	7 (1.71%)	19 (4.95%)	**0.010**

HG, Hungarian general population; HR, Hungarian Roma population. Problematic drinking: cutoff: men ≥8; women ≥5. Alcohol consumption at a hazardous level: scores of 1 or more on question 2 or question 3. Alcohol dependence: points scored above 0 on questions 4–6. Alcohol-related harm: points scored on questions 7–10. Past problems: based on the final two questions of AUDIT. ^*^Pearson's chi-squared and Fisher's exact test. Bold indicates statistical significance.

### 3.3. Multivariate analyses of alcohol consumption behaviors

Factors affecting alcohol use with regard to the studied indicators are shown in Table 5. Using multivariate logistic models that controlled for age, gender, education, marital status, financial status, and economic activity, Roma ethnicity was found to be a risk factor in two indicators related to alcohol consumption behavior. Compared to the Hungarian general sample, alcohol-related harm was three times higher (OR: 3.47; 95%CI = 1.61–7.49) in the Roma sample, and the Roma ethnicity was a significant risk factor (OR = 4.09; 95% CI = 1.02–16.46) for having past alcohol problems (Table 5).

**Table 5:** Results of multivariate negative binomial and logistic regression analyses on alcohol use as measured by AUDIT questions, with socioeconomic factors and ethnicity as the primary independent variables under investigation.

**Characteristics**	**Variable**	AUDIT OR^*^(95% CI)	Problematic drinking OR^**^(95% CI)	Hazardous level OR^**^(95% CI)	Alcohol dependence OR^**^(95% CI)	Alcohol-related harm OR^**^(95% CI)	Past problems OR^**^(95% CI)
Ethnicity	HG	1.00	1.00	1.00	1.00	1.00	1.00
	HR	1.29 (0.97–1.73)	1.69 (0.71–4.04)	1.14 (0.68–1.90)	1.29 (0.46–3.59)	**3.47 (1.61–7.49)**	**4.09 (1.02–16.46)**
Education	Primary or less	1.00	1.00	1.00	1.00	1.00	1.00
	Secondary	1.09 (0.80–1.48)	1.22 (0.51–2.91)	1.10 (0.65–1.88)	0.74 (0.25–2.21)	0.96 (0.44–2.11)	1.00 (0.26–3.82)
	High school	0.98 (0.70–1.39)	0.70 (0.21–2.32)	0.83 (0.45–1.54)	0.57 (0.15–2.21)	1.17 (0.46–2.99)	1.31 (0.24–7.24)
	Tertiary education	1.13 (0.75–1.72)	NC	1.01 (0.48–2.13)	0.24 (0.03–2.24)	0.61 (0.15–2.48)	NC
Economic activity	Worker	1.00	1.00	1.00	1.00	1.00	1.00
	Pensioner, other allowance, student	1.02 (0.77–1.36)	0.83 (0.31–2.22)	0.63 (0.35–1.14)	1.03 (0.33–3.21)	0.65 (0.26–1.62)	0.72 (0.17–2.98)
	Unemployed	1.10 (0.82–1.48)	0.97 (0.40–2.40)	1.28 (0.75–2.18)	2.46 (0.95–6.42)	1.28 (0.63–2.60)	1.54 (0.52–4.53)
Marital status	Married	1.00	1.00	1.00	1.00	1.00	1.00
	Single	1.18 (0.91–1.52)	1.69 (0.76–3.76)	**1.57 (1.00–2.48)**	1.26 (0.47–3.34)	1.61 (0.86–3.03)	0.95 (0.29–3.16)
	Widow, divorced	1.03 (0.77–1.39)	1.03 (0.40–2.68)	1.16 (0.67–2.03)	1.13 (0.39–3.33)	0.86 (0.36–2.10)	0.78 (0.20–2.95)
Financial status	Good	1.00	1.00	1.00	1.00	1.00	1.00
	Satisfactory	1.11 (0.87–1.42)	0.89 (0.37–2.16)	1.33 (0.84–2.12)	0.51 (0.20–1.30)	0.95 (0.49–1.85)	0.41 (0.13–1.32)
	Bad	1.34 (0.98–1.83)	1.68 (0.64–4.43)	1.38 (0.77–2.48)	0.87 (0.30–2.48)	1.24 (0.56–2.74)	1.32 (0.42–4.16)
Age category (years)	20–34	1.00	1.00	1.00	1.00	1.00	1.00
	35–49	1.08 (0.83–1.42)	1.73 (0.66–4.55)	0.73 (0.45–1.18)	1.88 (0.54–6.55)	0.85 (0.43–1.68)	3.94 (0.79–19.68)
	50+	0.98 (0.73–1.31)	1.70 (0.61–4.74)	**0.55 (0.32–0.93)**	3.13 (0.88–11.09)	0.86 (0.41–1.81)	4.06 (0.77–21.29)
Gender	Men	1.00	1.00	1.00	1.00	1.00	1.00
	Women	**0.27 (0.22–0.33)**	**0.30 (0.16–0.60)**	**0.18 (0.12–0.26)**	**0.11 (0.04–0.26)**	**0.14 (0.08–0.25)**	**0.12 (0.04–0.32)**

HG, Hungarian general population; HR, Hungarian Roma population; AUDIT, AUDIT total scores; OR, odds ratio; CI, confidence interval. ^*^Negative binomial regression. Problematic drinking: based on AUDIT scores, where cutoff: men ≥ 8. Women ≥ 5. ^**^Binary logistic regression. Alcohol consumption at a hazardous level: scores of 1 or more on question 2 or question 3. Alcohol dependence: points scored above 0 on questions 4–6. Alcohol-related harm: points scored on questions 7–10. Past problems: based on the final two AUDIT questions. NC, not countable due to low strata-specific numbers. Bold indicates statistical significance.

Furthermore, respondents' data showed a decrease in the risk of all analyzed outcomes (AUDIT total scores, problematic drinking, consumption at hazardous levels, alcohol dependence, alcohol-related harms, and past problems) for women, the oldest age category (50 years and older) decreased, and single marital status increased the risk of consuming alcohol at hazardous levels (Table 5).

The predicted ethnic differences in the prevalence of alcohol-related harm have been decomposed using B-O decomposition for non-linear models. As shown in Table 6, single marital status accounted for −7.90% of the alcohol-related harm frequency gap while gender explained −59.86%, which means equalizing gender and marital status explained differences would be expected to reduce the HG and HR alcohol-related harm frequency gap by about 8 and 60%, respectively. All of the coefficient effects were insignificant, which indicates the protective or risk effects of the studied variables are as strong for HG as they are for HR. Roma ethnicity has a significant positive effect (13.32 percentage points) on alcohol-related harm frequency. In other words, had ethnicity had no effect, the outcome gap would have been 89.24% lower. In the case of past alcohol problems, the decomposition could not be performed due to low stratum-specific numbers (only seven respondents were identified as having had past alcohol problems in the HG sample).

**Table 6:** Multivariate decomposition of the group (HG, HR) difference in alcohol-related harm.

		**Endowments**		**Coefficients**	
		**Coef. (95% CI)**	**Pct**.	**Coef. (95%CI)**	**Pct**.
	Primary or less	1.00			1.00
Education	Secondary	0.58% (1.90%; 3.05%)	8.18%	−0.22% (−3.56%; 3.13%)	−3.07%
	High school	−3.82% (11.33%; 3.69%)	−54.26%	2.58% (−3.47%; 8.64%)	36.07%
	Tertiary education	NC	NC	1.06% (−1.23%; 3.34%)	14.99%
	Worker	1.00			1.00
Economic activity	Pensioner, student	−0.14% (−0.43%; 0.15%)	−2.00%	−0.96% (−3.45%; 1.54%)	−13.59%
	Unemployed	1.43% (−0.75%; 3.60%)	20.25%	0.00% (0.00%; 0.00%)	0.00%
	Married	1.00			1.00
Marital status	Single	**−0.56% (1.01%; 0.10%)**	**−7.90%** ^*^	2.57% (−0.19%; 5.33%)	36.48%
	Widow divorced	−0.02% (−0.08%; 0.05%)	−0.23%	1.76% (−0.88%; 4.40%)	24.98%
	Good	1.00			1.00
Financial status	Satisfactory	0.02% (−0.30%; 0.34%)	0.28%	−0.35% (−6.15%; 5.45%)	−4.92%
	Bad	0.43% (−2.30%; 3.17%)	6.17%	−0.45% (−1.90%; 1.00%)	−6.37%
	20–34	1.00			1.00
Age category	35–49	0.05% (−0.08%; 0.17%)	0.68%	−0.07% (−4.50%; 4.36%)	−0.99%
	50+	−0.04% (−0.44%; 0.36%)	−0.53%	2.78% (−2.05%; 7.60%)	39.43%
Gender	Men	1.00			1.00
	Women	**−4.21% (5.94%; 2.49%)**	**−59.86%** ^**^	3.98% (−1.75%; 9.70%)	56.48%
Subtotal		−6.28% (15.37%; 2.80%)	−89.24%	**13.32% (3.00%; 23.64%)**	**189.24%** ^*^
Total	7.04% (3.00%; 11.08%)^**^			

HG, Hungarian general population; HR, Hungarian Roma population. Alcohol-related harm: points scored on questions 7–10. Endowments: due to differences in characteristics. Coefficients: due to differences in coefficients. ^*^p < 0.05, ^**^p < 0.01. Coef. (%), coefficients multiplied by 100; CI, confidence interval; Pct., expressed as a percentage; NC, not countable due to low strata-specific numbers. Bold indicates statistical significance.

## 4. Discussion

Globally, the highest levels of alcohol consumption are recorded in the WHO European Region, along with the greatest proportions of total ill health and premature death due to alcohol as well (6). Still, this burden is unevenly distributed among countries in Europe and for certain groups and ethnicities within countries, though not all countries have alcohol consumption and related data stratified by factors beyond age and gender (9). Even though, according to the WHO's Global Status Report on Alcohol and Health, alcohol consumption has declined in Hungary since and is expected to do so in the future, considering the prevalence of alcohol use disorders and dependence, the country still has one of the highest values in the world and among OECD countries (6, 8).

Since not only consumption levels but patterns are also responsible for alcohol-related harm, we aimed to collect data on alcohol consumption behavior with the comprehensive 10-question alcohol harm screening tool AUDIT in both the Hungarian general and Roma populations, the latter being the largest and most disadvantaged ethnic minority in Europe and Hungary, too. To the best of our knowledge, this is the first study in Hungary to collect alcohol consumption data from the Roma population with the AUDIT questionnaire and apply a decomposition analysis for the predicted ethnic differences in the prevalence of alcohol-related harm.

From an international point of view, studies using the same methodology are not common, may not be from the same period, and may not necessarily include individuals within the same age range. International studies available in the literature applying the AUDIT tool did not always use the same outcome and independent variables, and only a few of them analyzed alcohol-related harm. The AUDIT 10-item questionnaire was applied in four waves of the National Drug Strategy Household Survey (NDSHS) in Australia with respondents aged 14 years or older, analyzing total AUDIT scores, hazardous drinking (cut-off total score of 8 or more for both men and women), and AUDIT risk levels with independent variables of age and gender. In 2016, the percentage of men engaging in hazardous drinking was 14.99% (66) (HG: 7.39%; cut-off score 8 or more). According to the latest data available, recorded alcohol consumption among the population aged 15 and over was lower in Australia compared to Hungary (8). The lower proportion of HG men with problematic drinking may be explained by the methodology of the research itself. Higher levels or unhealthy patterns of alcohol consumption are sensitive issues, and since questionnaires were administered by practice nurses instead of GPs, underreporting could potentially affect our results. Other findings were in line with our research, i.e., men consumed more alcohol than women, and older age categories could be characterized as having lower risks for hazardous drinking (reference 18–24 years) (66).

Within the European context, Swedish data are available from the years 1997, 2001, 2005, 2009, 2014, and 2018 with equal proportions of men and women (aged between 17 and 80 years) included in the study. Subjects were contacted by letter; participation was anonymous and voluntary. Total AUDIT scores were described with respect to age and gender. Consistent with the literature and with our findings, men scored higher than women (67).

Studies assessing the alcohol consumption habits of the general population in Hungary with the same methodology are scarce and even conducted in different time domains. Our results about the Hungarian general population's alcohol consumption patterns may be compared with the findings of the Adult Population National Survey on Addiction Problems in Hungary (NSAPH 2015). This research was carried out on a nationally representative sample (2,274 individuals) of the Hungarian adult population aged 18–64 years of age, providing information on drinking frequency, quantity, heavy drinking, drunkenness, family history of regular alcohol use, and outpatient and inpatient care due to alcohol using the AUDIT questionnaire, among others (68). This survey found a higher prevalence of past 12-month drinkers (total 74.2%; men 83.3%; women 66.6%) compared to our data (total 53.19%; men 70.95%; women 39.30%). Furthermore, 78.8% of respondents in the study (men 66.7%, women 88.4%) never experienced a heavy drinking pattern (≥6 drinks on one occasion), and 14.6% (men 22%, women 8.5%) consumed less than they did monthly (69), which is in line with our findings. The higher abstinence rates of our Hungarian study sample may be explained by the higher health consciousness of enrolled individuals. Since data collection required an additional visit to GPs, which may be indicative of higher compliance and a higher awareness and understanding of health issues. Furthermore, questionnaires were administered by practice nurses (in contrast with NSAPH), making alcohol consumption a potentially sensitive issue for patients belonging to the GP practices. It is also important to note that our survey covered two counties in northeast Hungary, which are part of the Northern Great Plain region, where abstinence rates were found to be one of the highest in the country (70); meanwhile, the NSAPH collected alcohol consumption at the national level and also 3 years earlier.

Other AUDIT studies on the Hungarian general population were only available well before our study, making comparability of alcohol consumption data difficult. An investigation between 1997 and 2002 as a part of the GENACIS project focused on nine European countries (Switzerland, Spain, the U.K., Sweden, Finland, the Netherlands, the Czech Republic, Iceland, and Hungary) using the AUDIT tool to assess alcohol consumption behaviors. Comparing these data with our research findings should be done with caution since age ranges and interviewing methods differed (telephone, postal, face-to-face, self-administered) in the countries, and data collection took place much earlier than our investigation. Furthermore, types of alcohol-related harm were analyzed separately, rather than as a single outcome variable. In all countries, drinking frequency and quantity were higher for men. In 2001, as a part of this project, the alcohol behavior of the Hungarian sample (age range of 19–65 years) was analyzed with self-administered questions. In this European study, only Sweden and Iceland could be identified by a lower prevalence of drinking more than two drinks per week, and Hungarian women were characterized by the lowest frequency in this aspect. Although only Finland and Iceland preceded Hungary in terms of heavy drinking (at least once per month) among men, Sweden, Finland, and Iceland were characterized by a higher prevalence among women. When comparing the Hungarian data with our results, the frequency of drinking more than two drinks per week was similar, but the frequency of heavy drinking was found to be higher (71) when compared to our findings. Though these data would be hardly comparable with ours, as data collection occurred over one and a half decades apart, from 2000 to 2001 an increase was observed in alcohol consumption levels in Hungary (and stagnating at this level until 2006) and ever since, a general declining trend in consumption levels can be observed (72).

Comparing the alcohol behavior of the Hungarian general and Roma populations in our study suggests that although Roma people consume alcohol at lower frequencies, they experience more alcohol-related harm, even when considering past problems. This is congruent with the findings from Slovenia by Zelko et al. which is the only research available on Roma using the AUDIT questionnaire (49). Furthermore, studies in Europe also indicate that alcohol-related harm affects certain groups disproportionately, with the harm increasing with lower socioeconomic status, though mostly accompanied by lower consumption levels (9). Likewise, in the United States, alcohol use is also differentially associated with negative outcomes for different ethnic groups, which are not only related to the levels of alcohol consumption, since residents of socially disadvantaged areas and some ethnic minorities experience more harm per gram of alcohol consumed than those living in better conditions. The underlying causes may include differences in social and socioeconomic factors (73, 74), cultural differences in attitudes toward alcohol (74–76) and help-seeking behaviors, a shortage of health and social services, knowledge of negative consequences, shame and stigma associated with alcohol problems, and biological or genetic differences in alcohol metabolism (74).

Understanding the underlying social, cultural, and other factors contributing to the disparities in alcohol-related harm is essential from the point of view of public health (77). Although higher socioeconomic status (SES) is usually linked to more favorable health behaviors, in the case of alcohol consumption, the link is not always so clear-cut. The frequency of alcohol use tends to be higher among individuals with higher SES, but when considering drinkers, larger quantities are consumed by people with low socioeconomic status (78). Furthermore, more alcohol-related harm is experienced by some groups in socially disadvantaged areas and by some ethnic minorities than by those in better conditions (74). Discrimination and stigma clearly impact stress throughout life and risky health behaviors, including alcohol use. Excessive alcohol use can be a way to cope with the stress of everyday life and with ethnic discrimination, as research from the United States suggests. While the relationship with the African American community is unclear, higher alcohol consumption can be linked to self-reported unfair treatment and racial discrimination among the Asian American and Latino communities (16). In addition, research suggests that there may be a larger stigma associated with alcohol-consuming women in certain cultures (77). Furthermore, the built environment (poor conditions, including less favorable building conditions, housing, water safety, and sanitation) was also found to be associated with indicators of heavy drinking (79). These factors—which are all relevant for Roma living in Europe and Hungary—cannot be ignored when addressing racial and ethnic disparities in alcohol-related harm. When it comes to sensitive issues like alcohol problems, where there is a feeling that it may increase stigma against them, Roma's previous negative experiences with health services and mistrust of medical professionals may be a barrier to help-seeking. Delay in seeking help may also be caused by the problem that Roma often try to hide their mental health problems from family and from community members, which is due to the fear of damaging the family's reputation. Health treatment is often only sought at more advanced stages of the problem (80).

Cultural norms and beliefs toward alcohol consumption could be different and influence alcohol consumption patterns. Certain cultures may be permissive, which may apply to Roma as well because of a lack of knowledge of health consequences (74–76, 80). Besides a poor understanding of the nature of addiction itself (74–76, 80) and the availability of services and treatment options, Roma could be distrustful of the medical system (81). Physical barriers to healthcare services may delay or hamper receiving adequate medical care and counseling, especially in the case of Roma living in segregated colonies.

Our decomposition analysis revealed that, when considering alcohol-related harm, disparities in gender and relationship status affect Roma more than non-Roma, i.e., being single or a woman causes more intense differences. Equalizing the gender and marital status differences would be expected to reduce the HG and HR alcohol-related harm frequency gaps by about 8 and 60%, respectively. These findings might be explained by factors arising from Roma culture. This in fact means that, again, being single and a woman are considered inferior statuses in Roma communities (24, 73, 82–85), which may also contribute to differences in alcohol-related harm among them.

In addition to ethnic-specific differences, our study findings were consistent with common knowledge of the protective effect of being a woman on alcohol consumption behaviors, e.g., women are less likely to experience alcohol consumption at hazardous levels, suffer from the presence or incipience of alcohol dependence, and experience present or past alcohol-related harm (69, 71), even when dependence and alcohol consumption are measured with different tools (70, 86).

Some limitations of our study are related to the Roma population. The Roma sample was not representative of the overall Hungarian Roma population; it was only representative of Roma living in segregated colonies in northeast Hungary, where they are concentrated. Roma individuals who were, to various degrees, assimilated with the country's general population were not included in the analysis. Many Roma can be reluctant to self-define themselves as Roma (87); therefore, the reference sample of the Hungarian general population may have included some Roma people as well. It also has to be indicated that in our present study, the representation of women among HR was higher when compared to HG, similar to our previous surveys conducted among segregated Roma colonies in Hungary (88) and to a cross-sectional study conducted in Slovakia as well (89). This is because data collection occurred during the day when women were at home and men were away working. Between 2010 and 2015, the budget for public works was quadrupled by the Hungarian government for all Hungarian municipalities. This is especially relevant for villages situated in northeast Hungary, where segregated Roma settlements are concentrated, since the majority of participating workers in the program are men from underprivileged Roma communities (55). In addition, subjects aged 65 or over are not represented in our study samples. Since the representation of people over 65 years was as low as 3–4% in our previous Roma surveys (29, 53, 88), the size of the strata 65-X would be too small to make reliable conclusions for this subgroup of the population. Another limitation can arise due to the decreased number of Roma respondents in some sociodemographic strata (e.g., tertiary education). The small number of Roma subjects likely resulted in a type II error, which is responsible for the lack of observable differences between the Roma and non-Roma samples.

Furthermore, alcohol consumption and related problems may be underreported (90) even when using the AUDIT questionnaire (91), and the performance of the AUDIT screening tool may be diminished in certain ethnic minorities and ethnic groups with low acculturation (61). This problem may also occur in Roma communities since previous research suggests that Roma may be more inclined to please the investigators than the rest of the population, which may have an impact on the questionnaire results (76, 92, 93). This is especially relevant when asking about sensitive issues such as alcohol consumption. Roma people are already facing discrimination, stigma, and negative stereotypes and may fear answering alcohol consumption questions truthfully, which may be even more pronounced when describing the negative effects of alcohol use. The accurate understanding of the AUDIT questions and recalling the necessary information can also be a problem in some cases. However, these two potential issues were addressed by including Roma students as interviewers.

Nevertheless, investigating alcohol-attributed damage at the ethnic level provides important information to identify high-risk groups, and thus to design and implement more targeted and accessible therapies for alcohol problems. Our study provides insight into the alcohol consumption habits of the Hungarian general and Roma populations, pointing out a specific need for intervention targeting Roma people.

## 5. Conclusion

Our findings suggest that there are disparities in certain aspects of alcohol–related damage in Hungary when the Roma population is considered. Fighting stigma and poverty in the case of marginalized people, i.e., Roma living in European countries and Hungary, is essential to reducing inequities in alcohol-related harm. Furthermore, understanding the impact of culture on alcohol consumption is important for policymakers; culture-specific approaches are needed so that interventions and treatment options can meet the needs of vulnerable groups and ethnic minorities. Health education on the adverse effects of alcohol should be delivered and specifically tailored to Roma and removing barriers to receiving adequate health services is also essential. An option may be the improvement of screening and counseling (and treatment referrals when needed) delivered at the primary care level since GP practices are the most easily accessible for marginalized and segregated populations.

## Data availability statement

The original contributions presented in the study are included in the article/supplementary material, further inquiries can be directed to the corresponding author.

## Ethics statement

The studies involving human participants were reviewed and approved by the Ethical Committee of the Hungarian Scientific Council on Health (61327-2017/EKU). The patients/participants provided their written informed consent to participate in this study.

## Author contributions

AAMK was involved in data analysis and writing the manuscript. FV analyzed the data. PP took part in the creation of the database and the coding and sorting of the data. ZK and JS were involved in the design of the complex comparative health survey and data collection. RÁ took part in all steps of the development of the complex comparative health survey, guided the writing of the manuscript, and was involved in finalizing it. JD took part in interpreting the results and writing and finalizing the manuscript. All authors contributed to the article and approved the submitted version.
